# Code Similarity Prediction Model for Industrial Management Features Based on Graph Neural Networks

**DOI:** 10.3390/e26060505

**Published:** 2024-06-09

**Authors:** Zhenhao Li, Hang Lei, Zhichao Ma, Fengyun Zhang

**Affiliations:** School of Information and Software Engineering, University of Electronic Science and Technology of China, Chengdu 610054, China; hlei@uestc.edu.cn (H.L.);

**Keywords:** graph neural networks, multi-graph interaction, code feature, code similarity

## Abstract

The code of industrial management software typically features few system API calls and a high number of customized variables and structures. This makes the similarity of such codes difficult to compute using text features or traditional neural network methods. In this paper, we propose an FSPS-GNN model, which is based on graph neural networks (GNNs), to address this problem. The model categorizes code features into two types, outer graph and inner graph, and conducts training and prediction with four stages—feature embedding, feature enhancement, feature fusion, and similarity prediction. Moreover, differently structured GNNs were used in the embedding and enhancement stages, respectively, to increase the interaction of code features. Experiments with code from three open-source projects demonstrate that the model achieves an average precision of 87.57% and an *F*0.5 Score of 89.12%. Compared to existing similarity-computation models based on GNNs, this model exhibits a Mean Squared Error (MSE) that is approximately 0.0041 to 0.0266 lower and an *F*0.5 Score that is 3.3259% to 6.4392% higher. It broadens the application scope of GNNs and offers additional insights for the study of code-similarity issues.

## 1. Introduction

Code-similarity detection techniques can provide very important support for software development reuse, vulnerability impact analysis, and even the analysis of software supply chain security. For example, the development of reusable components is facilitated by analyzing similar or high-frequency code fragments across multiple software codes. As another example, similarity detection can be used to quickly analyze the scope of the impact of code with a certain vulnerability across multiple software versions.

The existing similarity-detection techniques [1] primarily utilize code text, structure, syntax, and semantics comprehensively and have yielded tools such as Nicad, Moss, and code2Vec, among others [2,3]. Similar codes can primarily be categorized into four types [4]:

Type I—code that is identical, except for whitespace, code comments, and layout.

Type II—the code structure and syntax are exactly the same, except for identifier names, types, layouts, and comments.

Type III—the features of Type II are met but with statements of addition, deletion, or modification.

Type IV—the code has the same functionality and effect but is implemented through a different syntactic structure.

Existing tools [5] have demonstrated effective detection of similarity for Type I, II, and III. However, Type IV codes pose challenges as their similarity features are not directly expressed in textual features such as method names and variable names.

Industrial management software is primarily utilized within the production, manufacturing, and management scenarios of industrial enterprises. Its fundamental operational logic involves the coordination and scheduling of materials, components, equipment, employees, vehicles, fields, time, and other resources throughout the production and manufacturing processes. Systems such as ERP, MES, and WMS are commonly employed to fulfill these functions. The primary characteristics of these systems [6,7] at the code level are marked by a heightened degree of individualization, evidenced by several distinct features: non-uniformity in variable naming conventions, substantial disparities in function declaration styles, complexity inherent in engineering structures and the interplay between modules, and a relative dearth of system API invocations. Collectively, these attributes align with the defining traits of Type IV codes, thereby rendering the computation of code similarity within the domain of industrial management software more challenging.

When reviewing the principles of code similarity-related processing techniques, it is observed that they all essentially encompass four steps: code preprocessing, code middle-level structure transformation, structure similarity metrics, and similarity identification. This entails transforming the source code from a two-dimensional, sequential simple structure into a multidimensional, multilevel enhanced information structure through deep structure transformation or information attachment. Subsequently, similarity computation is performed using algorithms based on numerical vector computation and deep learning [8,9].

In order to achieve a greater expression and transformation space for features among complex objects, the graph neural network (GNN) [10,11,12] has been proposed as a novel approach to characterize natural objects, following synthesis and innovation in the graph information structure [13,14]. This method integrates mechanisms such as node, edge, feature sampling, and message passing [15]. Furthermore, to extract multi-scale local spatial features and to reduce training costs while capturing features at different levels of abstraction, the Graph Convolutional Neural Network (GCN), which borrows from Convolutional Neural Networks (CNNs) in local feature extraction and weight-sharing, has been proposed. In recent research [16], numerous improved and evolved GNN models have emerged, each containing common propagation, sampling, and aggregation modules, albeit employing different intermediate processing methods. Examples include spectral-domain GCN [17], spatial-domain GCN [18], and the Graph Attention Network (GAT) [19], which incorporates attention mechanisms. Simultaneously, GNN networks exhibit the ability to handle features of different granularities at various levels, such as nodes, edges, and global graphs [20]. These advantages of GNN enable the features used for Type IV code similarity computation to overcome the limitations of low dimensionality and sequential flows.

As industrial management software continues to be utilized, its code base grows in size and complexity. In this context, the computation of similarity for Type IV codes becomes crucial for addressing software reuse and maintenance. However, existing methods are more effectively applied to the similarity computation of Types I, II, and III codes (for example, through the use of techniques such as AST syntax tree extraction, Program Dependence Graph construction, and semantic analysis). When applied to calculate the similarity of Type IV codes, they still face two major challenges: first, the challenge of capturing the features of the code’s context, including both internal and external environments; and second, the difficulties in processing features and applying similarity judgment methods, as well as the lack of in-depth exploration of code features and their complex relationships. These challenges significantly restrict the precision of code similarity computation.

In this study, we introduce a GNN-based similarity prediction model designated as FSPS-GNN. The model encompasses, in terms of code features, both the structure of the project in which the code resides and the logical features of the code itself, represented as a combination of two subgraphs: the inner and the outer. Regarding the computational process, the model interacts with, enhances, and fuses the dual features of the outer graph (oG) and the inner graph (iG) through a four-stage process and finally outputs the prediction through a fully connected (FC) network layer. In Stage I, the model addresses the interaction of features between oG and iG, thereby expanding the feature space and articulating the impact at the graph level. Stage II concentrates on the enhancement of the feature of three subgraphs in preparation for their fusion in Stage III. We conducted numerous experiments under various conditions, utilizing code datasets from three distinct sets of open-source projects with a focus on parameter optimization and analysis. Ultimately, FSPS-GNN was compared with several existing methods and models for similarity computation, demonstrating its effectiveness.

The main contributions of this study are as follows:

(1) A summary of the novel features pertinent to the similarity computation of Type IV codes and the strategies for leveraging them is provided: this includes integrating the structural features of the project within which the code is situated and the logical features inherent to the code itself.
(2) We propose a similarity-computation model that amalgamates the aforementioned dual features: the model operates by considering the relationship between the project structure and the code, utilizing a diverse array of GNN architectures. This approach enhances the richness and depth of the code’s features.
(3) We conducted extensive experiments on three typical open-source codes from industrial management software to substantiate the effectiveness and merits of our model. Specifically, it broadens the application scope of GNNs and offers additional insights into the study of code-similarity issues.

## 2. Related Work

### 2.1. Code Features in Projects

Teixeira [21] introduces a mechanism to elevate the level of abstraction in source code analysis by establishing a mapping from source code to compiler AST nodes using language specifications. Talbot [22] proposes a scheme that transforms static code into a universal graphical format (TGraph), enhancing the visual comprehension of programs for learners in programming. Cerny [23] addresses the overall quality assurance of service architecture in cloud-native systems by highlighting microservices’ dependencies from a system architecture viewpoint using static code, aiding developers in evolution and change management. Schiewe [24] presents a novel approach to overcome the limitations imposed by a single language and its underlying structure, converting source code into an intermediate representation known as language-agnostic AST, which is beneficial for tasks such as security assessment, coding practices, and error detection. Abdelaziz [25] introduces a set of universal code extraction techniques that represent classes, functions, and key syntax in code as a knowledge graph, supporting various applications like program search, code comprehension, refactoring, error detection, and code automation. Wang [26] proposes a new learning framework aimed at vulnerability detection, capturing the syntactic, semantic, and procedural features of a program by connecting statements through relational edges to form training samples. The aforementioned methods leverage the intrinsic syntactic and structural characteristics of code for feature extraction, but they rarely consider the relationships and structured representations within the project structure where the code resides. Our approach integrates both the inherent features of the code and the characteristics of its project structure, transforming them into graph data structures that are easily processed by deep learning networks.

### 2.2. Feature Embedding of Graphs

The relationship between pre-training and fine-tuning in the training process of graph neural networks was explored in Lu’s research [27], which proposed a dual adaptation mechanism capable of integrating local and global features. The investigation conducted in Papp’s research [28] focused on the relationship between the differentiation and expressiveness of message passing in the neighborhood structure of a graph, further supporting the advantages of GNNs over ordinary neural networks in feature expression. An envelope invariant variational auto-encoder was proposed in Winter’s research [29], addressing issues related to node ordering and matching of focused features during the unsupervised learning of graphs. A no-parameter pooling operator, introduced in Gao’s research [30], was designed to allow for greater preservation of feature information in arbitrary graphs. The relationship between the influence of distributional features of edges on the enhancement and representation of graphs was examined in He’s research [31]. Furthermore, Ling [32] introduced a deep graph matching and search model (DGMS) based on graph neural networks, which not only captures more structural information about individual query text or code snippets but also learns fine-grained similarities between them through a semantic matching operation based on cross-attention, providing references for the fusion of features from natural language and machine language. Li [33] presents a community detection model that underscores the embedding of node attributes and community structures, demonstrating notable efficacy in community classification. Yi [34] employs GNNs to develop a spatial embedding module, capturing the topological relationships between bike-sharing stations to bolster the model’s fine-grained representation. Ali [35] introduces a spatiotemporal GNN with node- and edge-embedding layers that harness topological attributes to iteratively refine the latent feature representations of road networks, with observed enhancements in computational efficiency in relation to model size. Collectively, these methods ameliorate the expressive capacity, granularity, and feature enhancement of graph structures within the training process to varying extents. In our approach, the enhancement and fusion processes are adapted for multiple subgraphs and their interactions, thus being more specifically targeted to the structural characteristics of software projects.

### 2.3. Similarity Computation of Graphs

An efficient graph distance based on the new field of geometric deep learning was proposed in Riba’s research [36]. This method enhances the message-passing neural network to capture the graph structure, resulting in improved performance in graph distance computation. TaGSim, a “type-aware” graph similarity learning and computation framework, was introduced in Bai J.’s research [37]. It allows flexible estimation of the graph edit distance (GED) based on different editing types of graphs, thereby addressing the issue of high computational overhead associated with traditional GED methods. Additionally, models proposed in Bai Y.’s research [38] and Xu’s research [39], based on graph segmentation and the graph neural network, respectively, were developed to enhance computational performance while maintaining the effectiveness of similarity computation compared to GED computation methods. This enhancement is achieved through the incorporation of local features, attention mechanisms focusing on the fine-grained features of graph embedding, and other mechanisms. While the previously discussed methods adopt an estimation-based approach to balance precision with computational efficiency across the entire computation process, our method meticulously tailors these design principles to specific local regions, thereby effectively minimizing the decrease in precision.

A multilevel graph-matching network based on GNN has been implemented in Ling X.’s research [40]. This network is capable of synthesizing node-level and global graph-level features, comprising a node-matching network and a Siamese GNN for calculating the similarity between any pair of graph-structured objects in an end-to-end manner. In Zhang’s research [41], a hierarchical hypergraph-based similarity matching network i been proposed, introducing subgraph matching and hyperedge pooling operators to enhance results in graph classification and recognition problems. Tan [42] addresses the issue of overlooking edge feature learning in graph similarity computation by introducing a proposed DGE-GSIM model. This model, based on the dual graph embedding mechanism, achieves feature fusion through graph–graph interaction, node–node interaction, and node–graph interaction along three pathways, thereby further enhancing the effectiveness of graph similarity computation. Additionally, Liu [43] proposes an embedded relational inference network to further enhance the performance of graph similarity and extract features more comprehensively at both the graph and node layers. While the aforementioned methods are capable of conducting similarity computations across a range of graphs or levels with bearable resource consumption, they are limited in comparison to our method in terms of feature computation between single graph ensembles. Moreover, there is a lack of measures to limit features in local areas of the graph, which can easily cause feature interference when dealing with node features formed by code. In our method, we introduce the kn parameter for the outer graph and the GAT mechanism for the inner graph, allowing more beneficial features to participate in the model’s similarity computation.

## 3. Preliminaries

### 3.1. Traditional Similarity Computations

**(1) Statistically based computations:** In statistic-based judgment, given the judgment space S, there are M possible features arranged in a fixed order, and the similarity can be obtained by calculating the proportion of $M_{A}\cap M_{B}$ to $M_{A}\cup M_{B}$. This method is only applicable to simple fixed-length arrays or text.

**(2) Cost-based computations:** Furthermore, to compute the similarity between objects of variable length, it is necessary to introduce the method of cost evaluation. That is, when objects A and B are different, from the perspective of dynamic change, the cost (elements, positions, etc.) of the changes required to change from A to B as a proportion of the overall metric counts of A and B can be taken as the similarity degree. Examples include the commonly used edit distance (ED) method and the graph edit distance (GED) method [44] extended to the graph domain.

**(3) Vector space-based computations:** Moreover, when multiple or multidimensional features exist between two objects, it is necessary to further introduce vector spaces for similarity comparison across multiple dimensions based on the statistical features of the previous methods, such as commonly known cosine similarity algorithms, Euclidean similarity algorithms, dot-product similarity algorithms, and so on.

### 3.2. Code Features of Industrial Management Software

**Figure 1:**
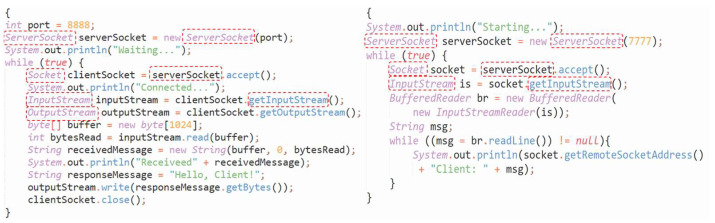
Example of similar codes for system-functional software (key features are indicated by red boxes).

**(1) Similarity features of Type II and Type III codes:** High precision in similarity computation is achieved when there is a distinct pattern of data objects and system API calls involved. As depicted in Figure 1, both code fragments implement a simple TCP connection. Despite some differences in programming style, overall code size, and variable naming conventions, there are statements that have been added, deleted, or modified within the code. Additionally, a significant number of system calls or built-in objects, such as **ServerSocket**, **InputStream**, and **OutputStream**, are present. Consequently, with the aid of textual features, weight assignment, and edit distance, the existing algorithms can still maintain a high level of usability for similarity computation.

**Figure 2:**
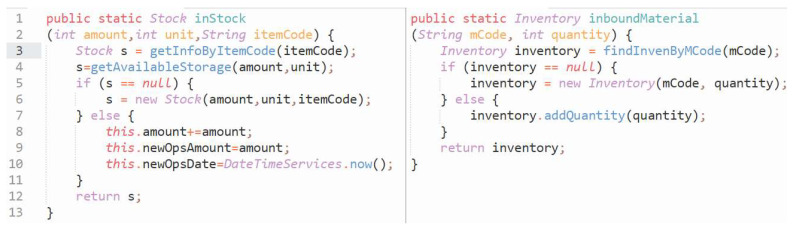
Example of similar codes used in industrial management software.

**(2) Similarity characteristics of Type IV codes:** For industrial management systems, codes that are equivalent or similar in terms of functional logic often exhibit a higher degree of custom-named variables or objects, and their syntactic structures can greatly differ. As illustrated in Figure 2, both codes are designed to serve the function of goods warehousing. However, due to different developers or development periods, the two codes significantly diverge in their naming conventions, code module divisions, and implementations of warehousing logic, with only some resemblance in the overall programming framework.

Such differences result in the near ineffectiveness of traditional methods that rely on keywords, syntax, and linear edit analysis for similarity computation. Furthermore, the structural composition of code features, as represented by the abstract syntax tree (AST) or program dependency graph (PDG), also varies considerably, which complicates the task of supporting similarity computation.

### 3.3. Properties of GNN

Scarselli’s research [10] on GNNs map the nodes in a graph to a Euclidean space. This mapping enables the generation of feature vectors for the nodes, which are used to represent the state of the nodes. It allows the model to utilize the neighborhood information of the nodes during loops and iterations, thereby achieving a richer representation of node features. Consequently, this leads to better handling of the complex interactions and relationships between nodes within the graph structure. Furthermore, this property of GNNs can reduce the dependence on the syntactic features of single lines of code when dealing with Type IV codes features. These attributes further support our adoption of learning mechanisms for processing complex codes and software engineering structures.

Scarselli posits that each node in a graph possesses a hidden state, which is updated during the learning process. At time *t* + 1, the hidden state $h_{v}^{t+1}$ of node v can be expressed by the following formula:

(1) $$h_{v}^{t+1}=f(x_{v},x_{e}\left(v\right),h_{ne}^{t}\left(v\right),x_{ne}(v))$$

where $x_{v}$ is a vector of features or attributes of the current node, $x_{e}\left(v\right)$ identifies the features or attributes of all edges connected to the current node, $h_{ne}^{t}\left(v\right)$ identifies the state of the neighboring nodes of the current node at the moment *t*, and $x_{ne}(v)$ represents the original features or attributes of the neighbors of the current node.

### 3.4. Preliminaries Summary

In summary, Type IV codes are more complex in terms of the variability of variable names, variability of statements, and variability of programming structure when compared to both fixed-length and indefinite-length texts and to general objects with multidimensional features. This complexity necessitates moving beyond superficial code text features when introducing new features and methodologies. When addressing code features, with the aid of graph information representation [45], we can encapsulate more nuanced features of the code in project structure as graph data structures, thus creating a richer feature space. Additionally, the characteristics of CNNs in terms of information transfer and implicit representation assist in constructing a predictive similarity-computation model.

## 4. Method

**Figure 3:**
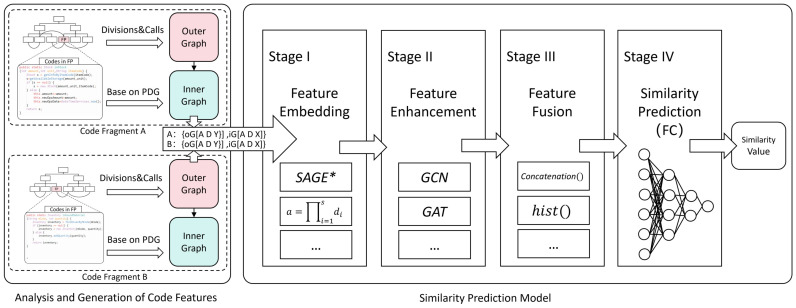
Overview of the processing of the method (view of steps).

The features of a piece of code within a development project can be derived from the code’s intrinsic features as well as from the structural features of the project itself. After representing these features using graph data structures, one can leverage the capabilities of GNNs for similarity computation. However, a challenge of this approach is the difficulty in generalizing controlled and sufficient rules due to the complexity of the feature semantics at the nodes and the interconnections of the edges. Therefore, we introduced learning and training mechanisms to form determination rules. Additionally, to mitigate the over-smoothing problem associated with GNNs, we also incorporated GCNs or related attention mechanisms. The focus of the method proposed in this study is on the feature embedding and enhancement of code, as well as the construction of a predictive network. As shown in Figure 3.

### 4.1. Embedding of Code Features

The code feature dimensions used for characterizing the code cannot solely focus on the textual semantics of objects and method names, as evidenced by previous observations of industrial management software code. Instead, these dimensions are influenced by both the overall architecture of the code project and the internal characteristics of the current function point (FP). Consequently, the similarity features of a code segment are divided into inner and outer parts. Inner features encompass adjacent lines of code within a function and a PDG serving as the foundational structure, with each line of program code representing a node and each set of adjacent lines and relationships in the PDG graph representing an edge. Outer features encompass the package path of the current module within the project (module division) and the calling relationships between modules, with each FP or module being a node and each set of modules or containment relationship between FPs being an edge.

Therefore, $X(x_{1},x_{2},x_{2})$ is defined as the vector of features of each node (each line of code) within the inner graph. $x_{1},x_{2},x_{3}$ represent the logical complexity level, the code nesting level, and the line number within a function, respectively. The logical complexity level is determined by the role that each line of code plays. In this paper, we define the complexity levels of the code in five distinct categories: function calls, mathematical operations, conditional judgments, declarations or initializations of variables and objects, and assignments. These levels are assigned values ranging from 5 to 1, respectively. Furthermore, $l_{\left(i,j\right)}$ is defined as the distance between two codes (which is used to form the adjacency matrix in Section 4.2), obtained from the difference between the actual line numbers in the codes. The features of iG of the code are referred to as such.

Meanwhile, $Y(y_{1},y_{2},y_{3})$ is defined the vector of features of each module (function) in the outer graph. $y_{1},y_{2},y_{3}$ represent the number of system API calls, the number of variables and objects, and table objects (by counting the codes within DAO layer associated with this module), respectively. $m_{\left(i,j\right)}$ is defined as the distance between two modules, which is equal to the reference strength QS between two modules, i.e.,

(2) $$m_{\left(i,j\right)}=QS=Q_{i,j}/\sum_{n=1}^{t}Q_{i,n}$$

**Figure 4:**
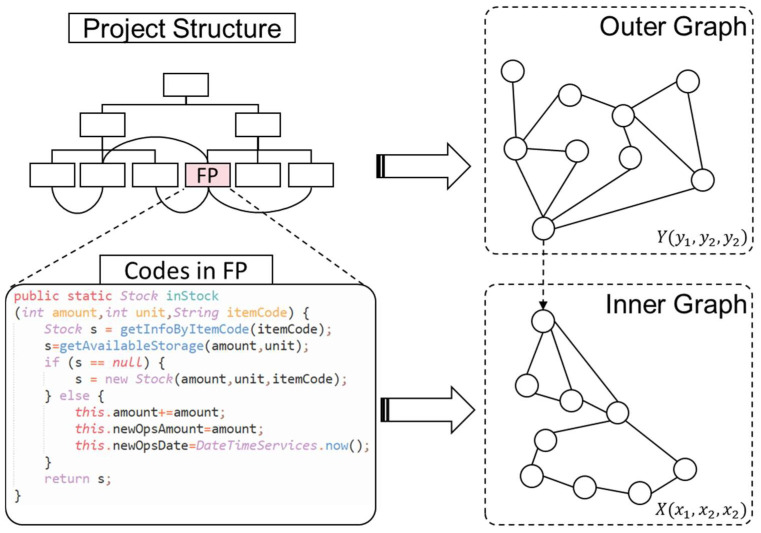
Example of the structure of the inner and outer graphs.

$Q_{i,j}$ is the one-time count of references (object generation, function calls, etc.) to module i by module *j*. *t* is the total number of modules in the project. The features of oG of the code is referred to herein. The inner and outer graphs are generated as depicted in Figure 4:

With the graph representation described above, the shortcomings of naming-based or text-based identification can be overcome, and instead, the features of the code structure and the role relationships between the modules in the project where the code is located can be highlighted. Feature data structured as a graph can also be constructed. Next, the methodology for constructing prediction model will be discussed.

### 4.2. Similarity-Prediction Model

In the problem addressed in this paper, two graph structures exist for each set of codes to be compared: the iG and the oG. These structures, characterized by varying levels of features, are interconnected. Relying solely on either of these graph structures to assess similarity is insufficient, and a mere numerical ratio judgment based (e.g., arithmetic averaging) on the similarity of the two pairs of graphs would overlook the interaction between the inner and outer graphs.

Consequently, the Father–Son Pairs Similarity GNN (FSPS-GNN) similarity-computation model is proposed, comprising four main components: feature embedding, feature enhancement, feature fusion, and similarity prediction. During the feature-embedding stage, we entered the inner and outer graph features of each code independently to form two feature sources and combined the interactions of the inner and outer graphs as a 3rd feature source. In the feature-enhancement stage, a combination of GCN and GAT networks was utilized for comprehensive message passing of the 3-way input features. Subsequently, during the feature-fusion stage, all processed features of the two codes were sampled and concatenated in a reasonable manner to derive the overall features post-fusion. Finally, in the similarity-prediction stage, the final output of the similarity prediction value was obtained via a FC layer neural network. The overall prediction of similarity ($S_{P}$) operational formula can be summarized as follows:

(3) $$S_{P}=FC\left(\sum fs(h^{\left(i\right)},h^{\left(o\right)},h^{\left(io\right)})\right)$$

**Figure 5:**
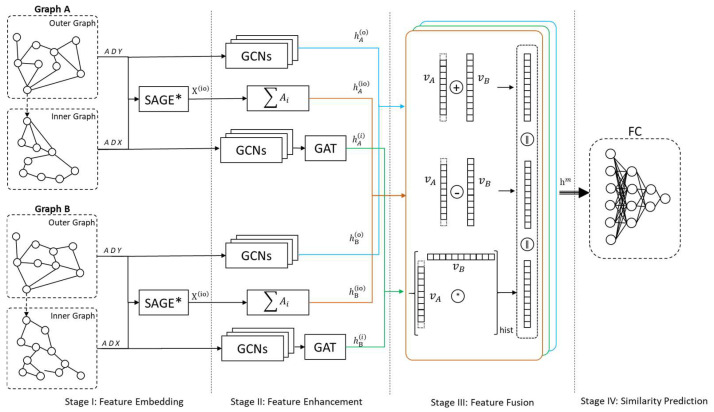
General structure of the similarity-prediction model.

The fs represents the feature fusion of Stage III in Section 4.2.3 below, and $h^{\left(io\right)},h^{\left(i\right)},h^{\left(o\right)}$ are computed by Formulas (4), (5) and (7) below, respectively. The structure of the whole model is shown in Figure 5:

#### 4.2.1. Stage I: Feature Embedding

In this stage, the inner structure graph iG and outer structure graph oG are directly passed forward in the network according to the feature-embedding in Section 4.1. The focus of this stage is to deal with the feature interactions between iG and oG, as shown in Figure 5. From the way iG and oG are associated and the adjacency matrix of oG, it can be seen that the closer the node is to the current module node (Nc), the more influence it has on Nc, and oG has a judgmental influence on the structural features of iG. Conversely, iG has no effect on oG. This unidirectional message delivery should follow some kind of range-decreasing law, while excessive feature amplification needs to be avoided when messages from oG are delivered to the inner part of iG, resulting in insufficient feature differentiation of iG. As shown in the first section of Figure 5, the adjacency matrix (A), degree matrix (D), and X/Y matrix of the subgraphs (inner or outer) of the two graphs under comparison were inputted into this network. A level-by-level-passing GraphSAGE [46]-like superposition structure was utilized to achieve feature interaction between them. Taking the input of one of the objects to be compared as an example, the following formula arises:

(4) $$X^{\left(io\right)}=\sigma [W\cdot X^{in}\cdot (X_{cv}^{ou}+OP(\sum_{k=1}^{k_{max}}A_{N\left(cv\right)}^{k}\cdot X_{{N\left(cv\right)}^{k}}^{ou}))]$$

In the above formula, $X^{in}$ is the feature matrix of iG, $X_{cv}^{ou}$ represents the feature vector of the node (current node) in the oG where iG is located, and *K-max* is the farthest neighbor distance of the current node; $A_{N(cv)}^{k}$ is the adjacency matrix formed by the distances from each node to the current node when the distance to the farthest neighbor is set to *k* in oG. If two nodes are reachable and the distance is s steps (traveling through s-1 nodes), the distance is $a=\prod_{i=1}^{s}d_{i}$, and $d_{i}$ is the distances from one node to the next node in the traveling; otherwise, a = 0. $X_{{N(cv)}^{k}}^{ou}$ is the feature matrix of the neighbor nodes with distance *k* from the current node. OP is the operator that performs the correlation operation on the aggregated information, and generally adopts the aggregation methods such as Sum, Mean, and Max.

#### 4.2.2. Stage II: Feature Enhancement

As depicted in Figure 5, the characteristics and structural features of three sets of input parameters determine their respective entry into distinct networks for feature enhancement. When processing the input of iG, as it represents the inner features of the function, these features have only initial and static relationships in the PDG structure in the representation, which has limited expressive ability. Therefore, the important features of the node are enhanced by combining the multilayer network of GCN and GAT. The input of oG, on the other hand, needs only feature enhancement based on the GCN network because it expresses the relationship between functions other than the current function, and its attention relationship is naturally established. The formula is as follows:

(5) $$h^{\left(i\right)}=\sum_{n=1}^{N}\left[\sigma (\sum_{l=1}^{l_{max}}\frac{1}{\sqrt{D^{l}}}A\frac{1}{\sqrt{D^{l}}}X^{\left(l\right)}W^{\left(l\right)})\cdot \left(h_{1}^{\prime }||\ldots ||h_{n}^{\prime }\right)\right]$$

(6) $$h_{i}^{\prime }=\sigma \left(\sum_{v_{j}\in N(v_{i})}\alpha_{ij}Wh_{j}\right)$$

(7) $$h^{\left(o\right)}=\sum_{m=1}^{M}\left[\sigma (\sum_{l=1}^{l_{max}}\frac{1}{\sqrt{D^{l}}}A\frac{1}{\sqrt{D^{l}}}X^{\left(l\right)}W^{\left(l\right)})+b_{cv}\right]$$

where *l* represents the number of layers of the GCN (generally 3 to 5 layers); *N* and *M* represent the total number of nodes in the iG graph and oG graph, respectively. The two sets of feature vectors, $h^{\left(i\right)}$ and $h^{\left(o\right)}$, can be obtained by summing the node-level features by rows, respectively. In the calculation of $h^{\left(o\right)}$, since the attention is naturally biased toward the current node cv, $b_{cv}$ is introduced here as an empirical parameter, and $b_{cv}$ can be obtained by taking the weights of the cv node in the D matrix and A matrix in oG as a reference. For $X^{\left(io\right)}$ entering Stage II, the $h^{\left(io\right)}$ feature vector is also obtained by summing the feature matrix by rows.

#### 4.2.3. Stage III: Features Fusion

For the $h^{\left(i\right)}$, $h^{\left(o\right)},$ and $h^{\left(io\right)}$ feature vectors obtained in the previous stage, two-by-two fusion between A and B via three sets of intermediate processes (yellow, green, and blue borders in Figure 5) results in three sets of feature vectors, and, finally, the three sets of vectors will be concatenated. At this stage, there are three pairs of feature vectors, $h_{A}^{\left(i\right)}$ and $h_{B}^{\left(i\right)}$, $h_{A}^{\left(o\right)}$ and $h_{B}^{\left(o\right)}$, and $h_{A}^{\left(io\right)}$ and $h_{B}^{\left(io\right)}$. Since these three pairs of vectors are processed in the same way, we replace $h^{\left(i\right)}$, $h^{\left(o\right)}$ and $h^{\left(io\right)}$ with v for the clarity of the internal operations.

Firstly, the vectors of the same pair are aligned according to the centering rule, the vector with longer length (e.g., with length P) is taken as the base, and the front and the back of the vector with a shorter length are complemented with zero elements of $\left|\left\|v_{A}\right\|-\left\|v_{B}\right\|\right|/2$ lengths, respectively. Then, the fused (*FS*) vectors of the two are computed and output. The formula is

(8) $$h^{m}=FS\left(v_{A},v_{B}\right)=\left(v_{A}+v_{B}\right)\;||\;\left|v_{A}-v_{B}\right|\;||\;({v_{A}}^{T}\times v_{B})$$

The three pairs of vectors of both *A* and *B* are computed using the above formula (|| in the formula represents vector concatenation), which results in three vectors, and finally, these three vectors are concatenated to obtain the final output vector ($h^{m}$) of Stage III. The HIST method refers to the histogram statistics of the matrix ℝ^p×p^ obtained from the ${v_{A}}^{T}\times v_{B}$ operation, where we take the minimum and maximum values in the matrix ℝ^p×p^ as the minimum and maximum values of the statistical range of the histogram. To keep the number of features aligned, the number of bins is set to P. Finally, the one-dimensional feature vector obtained by the histogram is normalized.

#### 4.2.4. Stage IV: Similarity Prediction

Based on the output vector $h^{m}$ from the previous stage, the prediction $S_{A,B}$ is output through a fully connected (FC) network with progressively decreasing number of node layers. This network is N-layered (generally taken as 4 to 8 layers), with the number of nodes reduced by 1/N at each layer, and the activation function uses the sigmoid function, where the formula is

(9) $$\sigma =\frac{1}{1+exp(-x)}$$

Finally, by comparing the predicted values with the ground-truth values (as described in Section 5.2), the loss function can be constructed. Specifically, to evaluate the model’s performance more comprehensively, this study employs two types of loss functions: Cross-Entropy Loss (*CEL*) and the Mean Squared Error (*MSE*):

(10) $$L_{CEL}=-\left(S^{g}\left(G_{A},G_{B}\right)\log\left(S_{A,B}\right)+\left(1-S^{g}\left(G_{A},G_{B}\right)\right)log\left(1-S_{A,B}\right)\right)$$

(11) $$L_{MSE}=\frac{1}{g}\sum_{i=1}^{g}{\left(S_{A,B}^{i}-S^{i}\left(G_{A},G_{B}\right)\right)}^{2}$$

where *g* is the number of batches during training, and $S^{i}\left(G_{A},G_{B}\right)$ is the ground-truth similarity value of the two codes.

### 4.3. Expression Space and Applicable Scenarios

Due to the lack of object name similarity in this problem scenario, the feature space of the code relies heavily on the (3 + 1) × (v + m) vector expression space jointly formed by graph iG and graph oG with node numbers v and w, respectively, before entering the model. After Stage I, the expression space consists of three parts: (3 + 1)v, (3 + 1)m, and (3 + 1) × max(v,m), with 2 × k_max + 2 linear transformations. During this stage, the emphasis lies on how features of graph oG are integrated into graph iG to an appropriate extent (avoiding over-smoothing). This integration encompasses both the appropriate distance relationships between neighboring nodes at various distances in graph oG and the proper output values after feature aggregation. At Stage II, the expression space is maximized in the instantaneous up to $\left(3+2\right)\times L[v^{2}+l_{max}\times (m+v)]$ when using a GCN layer parameter of L. Thus, after message passing and expansion in Stage I and Stage II, it is sufficient to augment the features of v + m nodes sufficiently. This is followed by Stage III, which outputs vector of length 3 × 3 × max(v,m) of obtained feature fusion and then FC network for prediction.

In summary, enhancements have been made to the model in terms of outer information fusion, information transfer between features, and expansion of the representation space through the four processing steps of the targeted design. The model is deemed suitable for similarity computation between two objects with limited expression of inner features, requiring supplementation by outer features. Besides the codes for industrial management features examined in this study, its applicability extends to similarity analysis in areas such as cross-system database tables and hybrid-structured transportation road networks.

## 5. Experimentation and Evaluation

### 5.1. Datasets

We selected three typical types of industrial management systems—ERP, MES, and WMS—as the subjects of our experiment. Each system type includes two distinct versions, resulting in a total of six systems sourced from the open-source communities on Github and Gitee. Specifically, these are JSH_ERP (JE) and FJ_ERP (FE), HM-MES (HM) and KTG_MES (KM), and LMH_WMS (LW) and JEE_WMS (EW), all of which are developed in Java, the primary programming language used for these systems.

### 5.2. Data Pre-Processing

We removed from the six system modules that solely express entity-relationship objects (such as Beans and DTOs) and generalized modules (e.g., logging, caching, and configuration modules) with minimal relevance to business functions, retaining only the logical function points (logical FPs) to form three datasets. In these datasets, the number of modules and the function points (FPs) for the systems are detailed in Table 1.

Each data sample is a code pair (cp), and the cp consists of logical FPs ($s_{label}$ or $d_{label}$) of two different systems ($S_{label}$ or $D_{label}$) in the same group. We analyzed the roles of annotations and the functional descriptions of Type IV codes using the principal current code structure analysis method [47] and classified the positive samples (same-effect FPs) from three datasets, labeled $S_{212}$, $S_{287}$, and $S_{310}$. In the meantime, we constructed negative samples (different-effect FPs), denoted as $D_{102}$, $D_{85}$, and $D_{87}$, based on the system from the same group that had a greater number of FPs in differential function points. To ensure the utilization of each negative sample, the codes from systems with fewer negative samples can be repeated in the code pairs up to a maximum number of times, which is a multiple of the difference in the number of negative samples between two systems within the same group. For clarity, the three datasets can be represented with the aforementioned notation:

(12) $${SET}_{JE\&FE}=\left\{\begin{array}{c} S_{212}=\left\{{cp\left[s_{JE},s_{FE}\right]}_{1}\ldots {cp\left[s_{JE},s_{FE}\right]}_{212}\right\}\;s_{JE}\in S_{JE},s_{FE}\in S_{FE}\\ D_{102}=\left\{{cp\left[d_{JE},d_{FE}\right]}_{1}\ldots {cp\left[d_{JE},d_{FE}\right]}_{102}\right\}\;d_{JE}\in D_{JE},d_{FE}\in D_{FE} \end{array}\right.$$

(13) $${SET}_{HM\&KM}=\left\{\begin{array}{c} S_{287}=\left\{{cp\left[s_{HM},s_{KM}\right]}_{1}\ldots {cp\left[s_{HM},s_{KM}\right]}_{287}\right\}\;s_{HM}\in S_{HM},s_{KM}\in S_{KM}\\ D_{85}=\left\{{cp\left[d_{HM},d_{KM}\right]}_{1}\ldots {cp\left[d_{HM},d_{KM}\right]}_{85}\right\}\;d_{HM}\in D_{HM},d_{KM}\in D_{KM} \end{array}\right.$$

(14) $${SET}_{LW\&EW}=\left\{\begin{array}{c} S_{310}=\left\{{cp\left[s_{LW},s_{EW}\right]}_{1},\ldots {cp\left[s_{LW},s_{EW}\right]}_{310}\right\}\;s_{LW}\in S_{LW},s_{EW}\in S_{EW}\\ D_{87}=\left\{{cp\left[d_{LW},d_{EW}\right]}_{1},\ldots {cp\left[d_{LW},d_{EW}\right]}_{87}\right\}\;d_{LW}\in D_{LW},d_{EW}\in D_{EW} \end{array}\right.$$

Additionally, due to the intrinsic structural variability inherent in Type IV codes, computed values based on the GED method [48] are no longer suitable as a reference. Consequently, we evaluated the effect of the function—considering function descriptions and parameters—as the ground truth for similarity, assigning a value of 1.0 for identical functionality and 0.0 for distinct functionality., e.g., checkInventory (String itemId, int quantity) vs. checkStockpiles (String materialId, int quantity) in the WMS system.

In the division of the training set, we combined the positive and negative sample sets from the three datasets and reordered the function names based on the total character length to enhance randomness—a common practice as developers often use an “operation + object” convention for naming, which resulted in related FPs being too close in the samples. Subsequently, we employed the k-fold cross-validation method, with the training set, validation set, and test set ratios allocated as 0.8:0.1:0.1, respectively. In this paper, the value of k is set to 5.

Furthermore, attention must be paid to the distribution of the graph (i.e., the iG) structures corresponding to the code within the datasets, with respect to both graph size and degree size, to ensure that the experiments are conducted on diverse and representative datasets.

**Figure 6:**
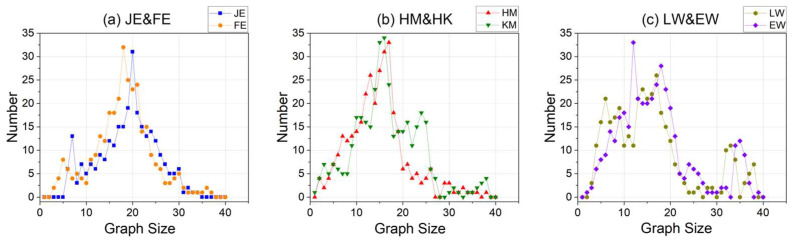
Distribution of the number of nodes in the iG for the three datasets.

**Figure 7:**
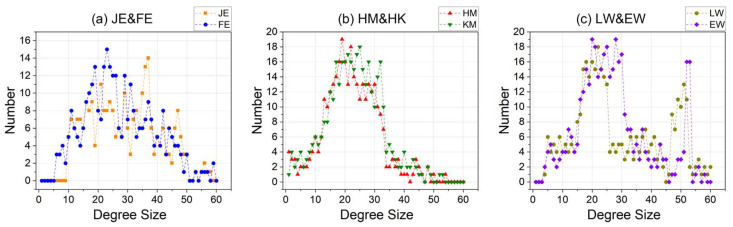
Distribution of the number of degrees in the iG for the three datasets.

For the three datasets, the iG size of the JE&FE dataset is predominantly centered around 20 (Figure 6a), while the degree size is concentrated between 15 and 40 (Figure 7a). The iG size of the HM&KM dataset is primarily concentrated around 15 (Figure 6b), with the degree size ranging between 15 and 35 (Figure 7b). Additionally, the iG size of the LW&EW dataset is mainly concentrated between 15 and 35 (Figure 6c), and the degree size is focused between 15 and 25, as well as between 45 and 55 (Figure 7c). It is evident that the three datasets exhibit significant differentiation in terms of graph structure and can support the experiment better.

### 5.3. Indicators for Evaluation

During the model’s training process, we utilized the aforementioned Cross-Entropy Loss (CEL) and Mean Squared Error (MSE) as crucial indicators to assess the training. Additionally, we considered the standard deviation (STD) and the confidence interval (CI) of the model across multiple experiments for reference. To evaluate the fundamental effectiveness of the model, we introduced the precision rate (*P*) and the recall rate (*R*) as metrics. Furthermore, given that the model’s application value lies in its ability to identify a greater number of code pairs with similarity, precision (*P*) holds greater importance over recall (*R*). Consequently, we employed the *F*0.5 Score as a complementary evaluation metric.

(15) $$F_{0.5}=1.25\cdot \frac{P\cdot R}{0.25P+R}$$

Additionally, for the purpose of distinguishing the characterization of the effect from the traditional approach, we converted the predicted similarity values into ranks after sorting them in descending order and obtained the correlation coefficients between the ranks and the ground-truth values based on two calculation methods, namely, Spearman’s correlation coefficient [49] (ρ), and Kendall’s correlation coefficient [50] (τ), which were used as references for the evaluation.

### 5.4. Baseline Parameters

For the framework parameters of the model, we made the number of layers of the GCN network lie between 2 and 6. To find the best settings, the size of each layer in the GCN was reduced by 1/2, the last layer was 16, and the activation function used ReLU. Given the coding specification, which typically limits the nesting of a function point to no more than 3 layers, the HEADS of the GAT network were set to 3, while a Dropout value of 0.2 was established. The batch size throughout the network remained constant at 128 for all stages. Additionally, the RMSprop algorithm [51] was employed to dynamically adjust the learning rate during the training process. Regarding the partitioning of the training set, a total of 212 pairs of positive and 102 negative samples were amalgamated. Subsequently, the training, validation, and testing set were allocated in a ratio of 0.8:0.05:0.15, respectively. The training and testing process was performed on three computers configured with E3 1275V3 CPUs, 64 GB of RAM, and NVIDIA V100 16 GB GPUs.

### 5.5. Experimental Results

#### 5.5.1. Model Performances and Comparisons

In addition to the parameter settings of the baseline, the model proposed in this paper allows for further tuning in the following three aspects, which can significantly impact the model’s performance: the scale of the oG, the number of layers in the GCN, and the computation of the aggregation operator within the SAGE* process. Baseline experiments were conducted with initial parameters set to layers = 3, k-nearest neighbors (kn) = 3, and Operator (OP) = Mean, and each dataset was evaluated using the k-fold cross-validation method. Subsequently, the best and worst experimental results from the multiple runs were discarded. The standard deviation (STD) and 95% confidence interval (CI) were then calculated to assist in the analysis. The experimental results are presented in Table 2.

As can be seen in Table 2, the model has shown preliminary effectiveness in predicting code similarity across the three datasets. When different loss functions are utilized, the precision (P) and *F*0.5 Score are marginally better with the CEL than with the MSE, although the difference is not substantial. However, it is notable that the time (T@80%) required with CEL is significantly less than with MSE when the model’s loss reduces to 80% of its initial magnitude. We attribute this to the larger gradient of CEL during training, which promotes quicker convergence.

We optimized by repeatedly adjusting the parameters of layers, k-nearest neighbors (kn), and Operator (OP). In the experiments (details are in Section 5.5.3), we confirmed that the optimal parameters for the model are layers = 3, kn = 3, and OP = Mean. Under these parameter settings, the optimized model demonstrates significant improvement over the baseline model to varying degrees, regardless of whether the CEL or MSE is utilized as the loss function. Specifically, the precision improves by approximately 4.2 to 8.02 percent, and the *F*0.5 Score improves by about 4.80 to 6.71 percent. Further details are presented in Table 3.

Additionally, the P and *F*0.5 Scores when utilizing the CEL loss function are slightly superior to those when using the MSE loss function. We attribute this to the MSE loss function’s susceptibility to gradient vanishing, particularly with this type of data labeling, during the final stages of model training, which can lead to less-comprehensive training. However, in general, the performance difference between using the CEL and MSE loss functions is minimal.

Furthermore, the model was compared with three classes of algorithms. The first class comprised GED-based algorithms, with the A*-Beamsearch (Beam) algorithm [52] and the HungarianGED algorithm [53] being selected, both of which are evolutionary algorithms derived from the A* algorithm and the Hungarian algorithm, respectively. The second category consisted of neural network algorithms based on classical parameter learning or attention mechanisms, namely, the SimpleMean and AttGlobalContext methods [54], respectively. The third category encompasses algorithms that leverage the nature of graph neural networks, and the SimGNN and DGE-GSIM methods were chosen for comparison. When conducting experiments with the aforementioned methods, the oG and iG integrated into a single graph in the object of study in this paper (depicted by the dotted line in Figure 4). Since the MSE is the common loss function employed in the aforementioned methods, we primarily focused on comparing the MSE and *F*0.5 Score. We used the average results from several experiments for this comparison, detailed in Table 4, Table 5 and Table 6.

In the comparisons, it is clear that the first type of traditional method based on the GED metric performs significantly worse than neural network methods in both MSE and *F*0.5 Score. In the case of the second type of neural network approach, its inability to simultaneously handle the interaction and fusion of node features and edge features limits its effectiveness in predicting the similarity of Type IV codes, which is a scenario with limited reliance on node features. In the third category of methods that employ GNNs, similarity prediction has seen significant improvement, with *F*0.5 Scores consistently above 80%. This enhancement stems from the GNN’s ability to capture not only the code itself but also the relationships between code segments, the context, and the intricate patterns within the code structure, providing richer and more comprehensive features less susceptible to minor variations. Building upon this, our method introduces an outer graph (oG) and an inner graph (iG) to further enrich the feature sources. By sampling through a SAGE-like mechanism controlled by “kn”, we generated post-interaction features (third feature source) for oG and iG that are well-suited to the structure of the software project. These features were then enhanced by integrating them with the architectures of both GCNs and GATs. As a result, our method achieves the best performance in Type IV code similarity prediction, outperforming existing GNN-based similarity-computation models with a lower MSE range of 0.0041 to 0.0266 and a higher *F*0.5 Score range of 3.3259% to 6.4392%.

#### 5.5.2. Ablation Experiment

To further confirm and validate the third source of validity of our model, a set of ablation studies were performed. In the third stage of the model, the $h^{\left(io\right)}$ introduced by the GAGE* method was removed, leaving only the two sets of feature inputs, $h^{\left(i\right)}$ and $h^{\left(o\right)}$:

(16) $$S_{p}^{\prime }=FC\left(\sum fs(h^{\left(i\right)},h^{\left(o\right)})\right)$$

Similarly, the ablation experiments were conducted on the three datasets.

The comparison results are presented in Table 7, Table 8 and Table 9, where red indicates optimal performance, blue signifies second-best, and green denotes third-best. Upon examining the aforementioned comparison parameters, it is evident that when the feature interactions of the outer graph oG and iG are omitted from our model, the model’s performance ranks between the second and third place, which is notably close to that of SimGNN. This suggests that the feature input represented by $h^{\left(io\right)}$ is not only valid but also highly significant.

#### 5.5.3. Model Parameters Analysis

To further understand the specific effects of the three parameters, namely, the size of the neighbor nodes of the outer graph (kn), the number of layers of the GCN network (layers), and the aggregation operator (OP), we analyzed the model’s performance under different parameters by using CEL and MSE as the observables. This analysis also reflects in more detail the process from the baseline to the optimized model in Section 5.5.1.

1. Size of neighbor nodes in the outer graph

**Figure 8:**
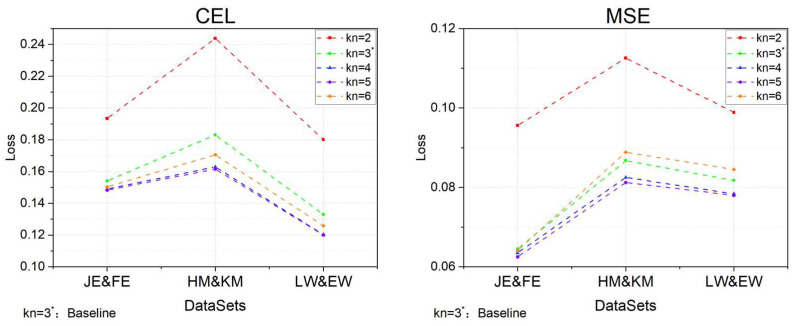
Performance of the model at different values of kn.

Given that the number of FPs in a software development project can range from several hundred to several thousand, the complexity of model training and computation escalates without constraining the node size of the oG. Moreover, as the number of nodes grows, FPs that are more distant from the current node increasingly exert a diminishing influence on information transfer. To address this, we introduced the kn value to denote the furthest node distance that can be integrated into the oG graph. In this experimental phase, the number of layers in the GCN was set to 3, and the SAGE process employed the Mean method as its aggregation operator. The experimental outcomes are depicted in Figure 8.

In the experiments conducted on the three datasets, both the CEL and MSE exhibited a significant downward trend as the model’s kn value increased from 2 to 4, with the smallest values occurring at kn = 5. However, when kn reached 6, CEL and MSE increased instead. Upon analysis, we determined that this is related to the common engineering structure of software, where the effective modules of the back-end logical structure typically consist of only 3 to 4 layers (e.g., com.product.subSystem.Module.function). In other words, while the number of neighbor nodes in the oG is increasing, the inclusion of structural information from FPs with little business relevance to the module can introduce feature interference into the oG, especially when kn exceeds 4. This can lead to a minor increase in MSE, as observed when kn = 6. Given that the computational complexity rises substantially with kn = 5 and the changes in CEL and MSE are minimal, considering all these factors, a kn value of 4 is deemed appropriate.

2. Number of layers in the GCN

**Figure 9:**
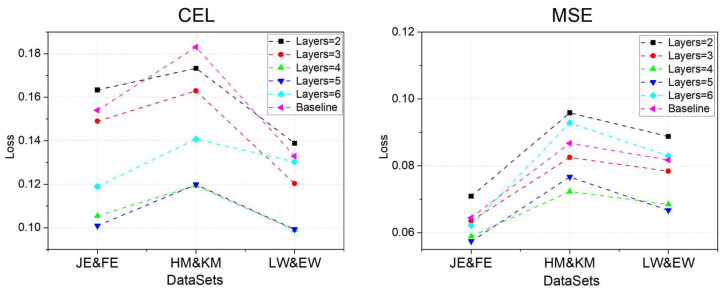
Performance of the model on different GCN layers.

Furthermore, with the settings fixed at kn = 4 and OP = mean, we investigated the impact of varying the number of layers in the GCN network on the model’s performance. The experimental results are presented in Figure 9.

In this set of experiments, we observed that the MSE continuously decreased as the number of layers in the GCN increased from three (as in the previous phase of experiments) to four layers. This suggests that an incremental increase in the number of layers aids in the enhancement of features. However, when the model’s GCN layer count was increased to five, the CEL and MSE were partially lower than when the layer count was four across six batches of experiments. Nevertheless, when the layer count increased to six, CEL and MSE markedly increased, even surpassing the baseline. Upon analysis, we determined that when the GCN layer count exceeds five, the feature vectors of the nodes start to converge toward the full graph, leading to feature convergence in both the oG and the iG, thereby exacerbating the over-smoothing problem. Hence, the number of layers should be capped at less than six. Considering the training time expenditure as well, we concluded that setting the number of layers to four is the most optimal choice.

3. Aggregation operator

**Figure 10:**
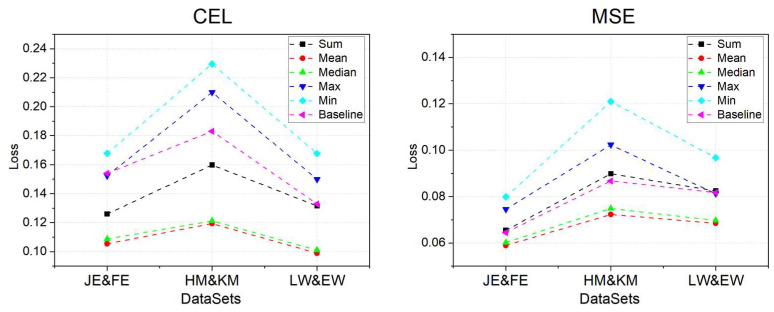
Performance of the model when different aggregation operators are used.

Finally, after determining the values for kn and the number of layers, we compared the effects on the model when different aggregation operators were utilized in the SAGE* process for both the iG and the oG. We experimented with a total of five aggregation methods: Sum, Mean, Median, Max, and Min. The experimental results are presented in Figure 10.

Among all the aggregation operators tested, the Mean operator performs the best, with the lowest CEL and MSE. Upon analyzing the data samples and outputs from intermediate steps, we found that the primary reason for the average performance of the Sum operator is its tendency to amplify the feature values in aggregations, which can lead the model into an over-smoothing state more rapidly. Moreover, since the feature gap values between nodes or edges in the oG and the iG are not consistent after sorting from largest to smallest, the Median operator is restricted to conveying a local feature that may not be representative, resulting in poorer performance compared to the Mean method. Additionally, both the Max and Min operators result in the loss of a significant number of features and thus perform the worst. Consequently, we ultimately used the Mean as the aggregation operator.

#### 5.5.4. Case Studies

**Figure 11:**
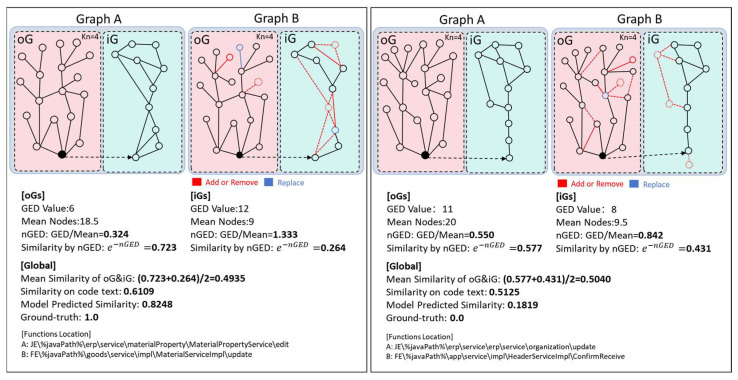
Cases of positive and negative examples.

To further demonstrate the utility and significance of the FSPS-GNN model, we selected two graph data samples—representing one positive and one negative code pair—from the datasets. These samples originate from the material function and the auxiliary management function of the ERP system within the dataset, as shown in Figure 11.

As seen in Figure 11 and its underlying data, from the perspective of GED-based similarity between code pairs, the oG contributes more similar features, whereas it is more difficult to combine the use of GED methods (e.g., the mean similarity of GED) to determine the similarity between oG and iG due to the large differences in the code styles of iG (the value of similarity is usually too close to 0.5 and difficult to distinguish). In addition, the textual features of Type IV codes are almost entirely unrelated to their similarity in terms of efficacy. The model proposed in this paper integrates deeper features of oG and iG through the inputs $h^{\left(io\right)}$, $h^{\left(i\right)}$, and $h^{\left(o\right)}$ and has a better ability to determine the similarity of Type IV codes.

### 5.6. Summary of Experiments

Through the aforementioned experiments, we conducted model experiments and optimizations on three open-source project datasets. The results have confirmed that our method exhibits significant improvements in terms of CEL, MSE, and *F*0.5 Score metrics when compared to existing, improved GNN-based methods. In particular, the *F*0.5 Score is 3.3259% to 6.4392% higher than that of other training-based models. Additionally, we demonstrated the necessity and effectiveness of the FSPS-GNN model for processing feature interactions of the oG and iG through ablation experiments. Finally, we further elucidated the characteristics of the model through a case study.

## 6. Conclusions and Future Works

In this study, we explored the structural characteristics of codes and projects within the domain of industrial management and introduced an innovative model, FSPS-GNN, designed for similarity computation of Type IV codes. The study’s core contribution lies in the integration of multiple GNN feature-transfer and fusion mechanisms, an innovation that markedly enhances the model’s capability to compute code similarity. We thoroughly validated the effectiveness of the FSPS-GNN model through extensive experiments across three distinct open-source projects, encompassing baselining, optimization, and comparative analyses. The experimental outcomes demonstrate that the FSPS-GNN model holds significant advantages in terms of MSE and *F*0.5 Score when juxtaposed with existing traditional neural network models, as well as other GNN models, particularly for the task of Type IV code similarity computation.

Furthermore, this study includes ablation experiments and case studies on the model’s key input features, which further elucidate the impact of different features on the model. This provides valuable insights and references for future research and applications in this field.

Overall, the FSPS-GNN model provides a new perspective for analyzing Type IV codes in industrial management software. Future work lies in the following areas:

(1) Further analyzing the characterization in depth and improving the model accuracy.
(2) Increasing the experimentation and analysis of Type IV codes in other programming languages.
(3) Optimizing computations at each stage of the model and reducing the model’s training time.

## Figures and Tables

**Table 1:** Number of modules and functions of the three experimental objects.

Dataset Groups	Group 1		Group 2		Group 3	
Projects	JE	FE	HM	KM	LW	EW
Total Modules	309	1171	834	845	607	799
FPs	692	766	423	489	502	527
Logical FPs	276	314	334	372	364	397
Same-effect FPs (S)	212	212	287	287	310	310
Different-effect FPs (D)	64	102	47	85	54	87

**Table 2:** Model’s baseline performance.

Metrics		CEL				MSE			
		${Loss}^{\left(10^{-2}\right)}$	$P^{\left(10^{-2}\right)}$	${F0.5}^{\left(10^{-2}\right)}$	T@80%(s)	${Loss}^{\left(10^{-2}\right)}$	$P^{\left(10^{-2}\right)}$	${F0.5}^{\left(10^{-2}\right)}$	T@80%(s)
JE&FE	Mean	15.3981	82.5472	84.4595	189.34	8.4072	82.1859	83.9768	423.83
	STD	0.0621	0.3880	0.7755	3.18	0.0431	0.4111	0.7320	3.27
	CI	±0.0253	±0.1586	±0.3169	±1.59	±0.0176	±0.1680	±0.2992	±1.34
HM&HK	Mean	18.3073	80.8362	82.7980	208.55	8.6755	80.5437	82.4246	583.12
	STD	0.0645	0.5923	0.3891	5.21	0.0607	0.7641	0.4025	6.26
	CI	±0.0264	±0.2421	±0.1590	±2.12	±0.0248	±0.3123	±0.1645	±2.56
LW&EW	Mean	13.2932	84.5161	85.2865	278.32	8.1750	83.5467	84.5641	676.85
	STD	0.0715	0.4262	0.4183	9.44	0.0512	0.4331	0.4127	11.13
	CI	±0.0292	±0.1742	±0.1709	±3.86	±0.0209	±0.1771	±0.1687	±4.55

**Table 3:** Optimized model performance.

Metrics		CEL			MSE		
		${Loss}^{\left(10^{-2}\right)}$	$P^{\left(10^{-2}\right)}$	${F0.5}^{\left(10^{-2}\right)}$	${Loss}^{\left(10^{-2}\right)}$	$P^{\left(10^{-2}\right)}$	${F0.5}^{\left(10^{-2}\right)}$
JE&FE	Mean	10.5419	90.5660(+8.02)	91.1681(+6.71)	5.6894	88.2075(+6.02)	89.8175(+5.84)
	STD	0.0502	0.3912	0.7877	0.0389	0.3756	0.6722
	CI	±0.0205	±0.1599	±0.3219	±0.0159	±0.1535	±0.2747
HM&HK	Mean	11.9418	88.5017(+7.67)	89.4366(+6.64)	7.2312	86.7596(+6.22)	88.1728(+5.75)
	STD	0.0621	0.5832	0.3742	0.05897	0.7338	0.3765
	CI	±0.0254	±0.2383	±0.1529	±0.0241	±0.2999	±0.1539
LW&EW	Mean	9.8927	89.3548(+4.84)	90.5821(+5.29)	6.8471	87.7419(+4.20)	89.3561(+4.80)
	STD	0.0702	0.4063	0.3988	0.04871	0.4206	0.3985
	CI	±0.0287	±0.1660	±0.1630	±0.0199	±0.1719	±0.1629

**Table 4:** Comparison of models using the JE&FE dataset.

Method	MSE (10^−2^)	*F*0.5 (10^−2^)	ρ	τ
Beam	33.0281	61.9374	0.5564	0.5273
Hungarian	35.2392	60.0112	0.5361	0.5141
SimpleMean	18.8653	76.0902	0.4021	0.3232
AttGlobalContext	8.3028	81.1079	0.7513	0.7275
SimGNN	7.8456	84.2307	0.8158	0.7812
DGE-GSIM	6.0947	86.3482	0.8371	0.8109
FSPS-GNN	**5.6894**	**89.8175**	**0.8687**	**0.8304**

**Table 5:** Comparison of models using the HM&HK dataset.

Method	MSE (10^−2^)	*F*0.5 (10^−2^)	ρ	τ
Beam	37.3439	59.3171	0.5277	0.5088
Hungarian	38.4955	58.2283	0.5016	0.4892
SimpleMean	19.9879	73.8912	0.3798	0.3163
AttGlobalContext	10.5526	78.5879	0.7290	0.7044
SimGNN	9.8924	80.3204	0.8003	0.7603
DGE-GSIM	8.4866	83.1743	0.8102	0.7975
FSPS-GNN	**7.2312**	**86.7596**	**0.8468**	**0.8172**

**Table 6:** Comparison of models using the LW&EW dataset.

Method	MSE (10^−2^)	*F*0.5 (10^−2^)	ρ	τ
Beam	35.3189	60.8773	0.5406	0.5146
Hungarian	37.2842	58.2183	0.5148	0.4997
SimpleMean	19.2351	73.8912	0.3976	0.3199
AttGlobalContext	9.7947	80.0141	0.7413	0.7183
SimGNN	9.0765	83.5487	0.8106	0.7767
DGE-GSIM	7.8653	86.0302	0.8236	0.8045
FSPS-GNN	**6.8471**	**89.3561**	**0.8578**	**0.8246**

**Table 7:** Comparison of ablation experiment on JE&FE. Red is first, blue is second, and green is third. * Indicates that the model modifies according to the ablation experiments described above.

Method	MSE (10^−2^)	*F*0.5 (10^−2^)	ρ	τ
Beam	33.0281	61.9374	0.5564	0.5273
Hungarian	35.2392	60.0112	0.5361	0.5141
SimpleMean	18.8653	76.0902	0.4021	0.3232
AttGlobalContext	8.3028	81.1079	0.7513	0.7275
SimGNN	7.8456 ^3^	84.2307 ^3^	0.8158 ^3^	0.7812 ^3^
DGE-GSIM	6.0947 ^1^	86.3482 ^1^	0.8371 ^1^	0.8109 ^1^
FSPS-GNN *	6.9112 ^2^	84.9541 ^2^	0.8194 ^2^	0.7993 ^2^

**Table 8:** Comparison of ablation experiment on HM&HK. Red is first, blue is second, and green is third. * Indicates that the model modifies according to the ablation experiments described above.

Method	MSE (10^−2^)	*F*0.5 (10^−2^)	ρ	τ
Beam	37.3439	59.3171	0.5277	0.5088
Hungarian	38.4955	58.2283	0.5016	0.4892
SimpleMean	19.9879	73.8912	0.3798	0.3163
AttGlobalContext	10.5526	78.5879	0.7290	0.7044
SimGNN	9.8924 ^2^	80.3204 ^3^	0.8003 ^3^	0.7603 ^3^
DGE-GSIM	8.4866 ^1^	83.1743 ^1^	0.8102 ^1^	0.7975 ^1^
FSPS-GNN *	9.9101 ^3^	80.3302 ^2^	0.8057 ^2^	0.7631 ^2^

**Table 9:** Comparison of ablation experiment on LW&EW. Red is first, blue is second, and green is third. * Indicates that the model modifies according to the ablation experiments described above.

Method	MSE (10^−2^)	*F*0.5 (10^−2^)	ρ	τ
Beam	35.3189	60.8773	0.5406	0.5146
Hungarian	37.2842	58.2183	0.5148	0.4997
SimpleMean	19.2351	73.8912	0.3976	0.3199
AttGlobalContext	9.7947	80.0141	0.7413	0.7183
SimGNN	9.0765 ^3^	83.5487 ^3^	0.8106 ^3^	0.7767 ^3^
DGE-GSIM	7.8653 ^1^	86.0302 ^1^	0.8236 ^1^	0.8045 ^1^
FSPS-GNN *	9.0232 ^2^	83.7851 ^2^	0.8144 ^2^	0.7802 ^2^

## Data Availability

Data are contained within the article.
